# The Gametic Non-Lethal Gene *Gal* on Chromosome 5 Is Indispensable for the Transmission of the Co-Induced Semidwarfing Gene *d60* in Rice

**DOI:** 10.3390/biology8040094

**Published:** 2019-12-17

**Authors:** Motonori Tomita, Takatoshi Tanisaka

**Affiliations:** 1 Laboratory of Genetics and Genome Engineering, Research Institute of Green Science and Technology, Shizuoka University, Shizuoka 422-8529, Japan; 2 Laboratory of Breeding, Faculty of Agriculture, Kyoto University, Kyoto 606, Japan; t_tanisa@kiui.ac.jp

**Keywords:** rice, complementary gamete lethal, non-Mendelian ratio, mapping, NGS, pollen development, pollen second mitosis

## Abstract

The gametic lethal gene *gal* in combination with the semidwarfing gene *d60* causes complementary lethality in rice. Here, we attempted to ascertain the existence of *gal* and clarify male gamete abortion caused by *d60* and *gal*. Through the F_2_ to F_4_ generations derived from the cross between *D60gal*-homozygous and *d60Gal*-homozygous, progenies of the partial sterile plants (*D60d60Galgal*) were segregated in a ratio of 1 semidwarf (1 *d60d60GalGal*):2 tall and quarter sterile (2 *D60d60Galgal*):6 tall (2 *D60d60GalGal*:1 *D60D60GalGal*:2 *D60D60Galgal*:1 *D60D60galgal*), which is skewed from the Mendelian ratio of 1 semidwarf:3 tall. However, the F_4_ generation was derived from fertile and tall heterozygous F_2_ plants (*D60d60GalGal*), which were segregated in the Mendelian ratio of 1[semidwarf (*d60d60GalGal*)]:2[1 semidwarf:3 tall (*D60d60GalGal*)]:1[tall (*D60D60GalGal*)]. The backcrossing of *D60Gal-*homozygous tall F_4_ plants with Hokuriku 100 resulted in fertile BCF_1_ and BCF_2_ segregated in a ratio of 1 semidwarf:3 tall, proving that *d60* is inherited as a single recessive gene in the *D60d60GalGal* genetic background (i.e., in the absence of *gal*). Further, *gal* was localized on chromosome 5, which is evident from the deviated segregation of *d1* as 1:8 and linkage with simple sequence repeat (SSR) markers. Next-generation sequencing identified the candidate SNP responsible for *Gal*. In F_1_ and sterile F_2_, at the binucleate stage, partial pollen discontinued development. Degraded pollen lost vegetative nuclei, but second pollen mitosis raising two generative nuclei was observed. Thus, our study describes a novel genetic model for a reproductive barrier. This is the first report on such a complementary lethal gene, whose mutation allows the transmission of a co-induced valuable semidwarfing gene *d60*.

## 1. Introduction

The “Green Revolution” of the 1960s, in which the production of grain was dramatically increased through the breeding and development of semidwarf varieties of rice and wheat, is probably the greatest agricultural contribution in the history of mankind. Semidwarfness prevents plants from lodging at their full-ripe stage, making them lodging-resistant to wind and rain, enhances their adaptability for heavy manuring, and markedly improved the global productivity of rice and wheat between 1960–1990 (up to double yields of rice and quadruple yields of wheat) [1]. The semidwarf “miracle rice” variety IR8 released by the International Rice Research Institute (IRRI) responds particularly well to fertilizer inputs and produces increased yields without culm elongation [2]. The widespread adoption of IR8 brought about a “green revolution” in the monsoonal regions of Asia, where typhoons frequently occur during the yielding season. In addition, semidwarfness brings benefits such as erect leaf angles, reduced photoinhibition, and the possibility of planting at higher densities. For this reason, semidwarf varieties were also introduced into California and also in Latin America [3].

Several dwarf genes have been isolated, but many of these dwarf phenotypes are the result of deficiencies in the gibberellin (GA) biosynthesis pathway, which controls the levels of GA1, a final product of active GA, in the stem and leaf. The *sd1* alleles, on the long arm of chromosome 1 [4,5,6], encode a defective C20-oxidase in the gibberellin (GA) biosynthesis pathway (GA 20-oxidase, *OsGA20ox2*) [7,8,9,10] and mutations in the GA20-oxidase gene lead to disruptions at a late stage of the GA pathway [7]. The *sd1* gene confers the semidwarf phenotype with no detrimental effects on grain yield [11,12,13]. Although semidwarf varieties of rice have contributed to the dramatic improvement and stabilization of yields worldwide, the semidwarf stature of varieties derived from native or mutant maternal lines happen to be controlled by a single gene, *sd1* [7,9,14,15,16], as it is an oligopoly condition of *sd1.* Both the Tanginbouzu *d35* and Kotake-tamanishiki *d18-k* genes are kaurenoic acid oxidase-defective or 3-beta hydroxylase-defective in the same GA biosynthesis pathway [17]. Other dwarf genes such as *d11* [18] and *sd37* [19], whose function is not related to the GA biosynthesis pathway, were certainly identified. However, their practical use in breeding has not yet proceeded. A little genetic source of current semidwarf rice cultivars has a risk for environmental change. Thus, it is necessary to acquire a wider range of semidwarfng genes to cope with future environmental changes.

In order to identify a novel alternative semidwarf gene to *sd1*, we conducted gene analyses focusing on Hokuriku 100, a mutant breeding rice strain with a 20% shorter culm than the Koshihikari variety. Hokuriku 100 was developed through a large-scale mutation breeding operation using ^60^Co irradiation to overcome the lodging weakness of Koshihikari [20]. The first author analyzed a mutation of Hokuriku 100 [21,22] and observed abnormal segregation in the ratio of 40 semidwarf:294 tall between Koshihikari and Hokuriku 100, which is skewed from the expected 1:3 ratio of the F_2_ population. The first author suspected that this might be attributed to the partial seed sterility of 25% in the F_1_ and some of the F_2_ tall plants. An F_3_ progeny test was conducted in which both semidwarfness and seed sterility were observed, and the following hypotheses were proposed: 1) Koshihikari carries a gametic lethal gene, *gal*; 2) Hokuriku 100 carries a gametic non-lethal gene, *Gal*, mutated from *gal*, as well as its activator, *d60*; 3) male and female gametes carrying both *gal* and *d60* are lethal. To date, there is no evidence that the supposed semidwarf gene *d60* is inherited as a single recessive gene according to the ratio of 1*D60D60:*2*D60d60*:1*d60d60*. However, double dwarfness due to a combination of *d60* and *sd1* was obtained via skewed segregation, and therefore *d60* is regarded as an independent allele of *sd1* [23].

The objectives of this study were: (1) to prove the existence of the supposed gametic lethal gene *gal* by genetic analysis of the skewed segregation of semidwarfness accompanied by seed sterility from F_1_ to F_4_ generations; (2) to confirm the Mendelian ratio of the *d60* allele in the genetic background of the gametic non-lethal allele *Gal* homozygous in F_4_ and BCF_1_; (3) to identify the chromosomal localization of *gal* by the deviated segregation of linked morphological markers, linkage analysis with DNA markers, and whole genome sequencing with next-generation sequencing (NGS); and (4) to clarify the male-gamete abortion caused by *d60* and *gal* through cytological observation.

## 2. Materials and Methods

### 2.1. Genetic Analysis of d60 and Gal 

**Figure 1:**
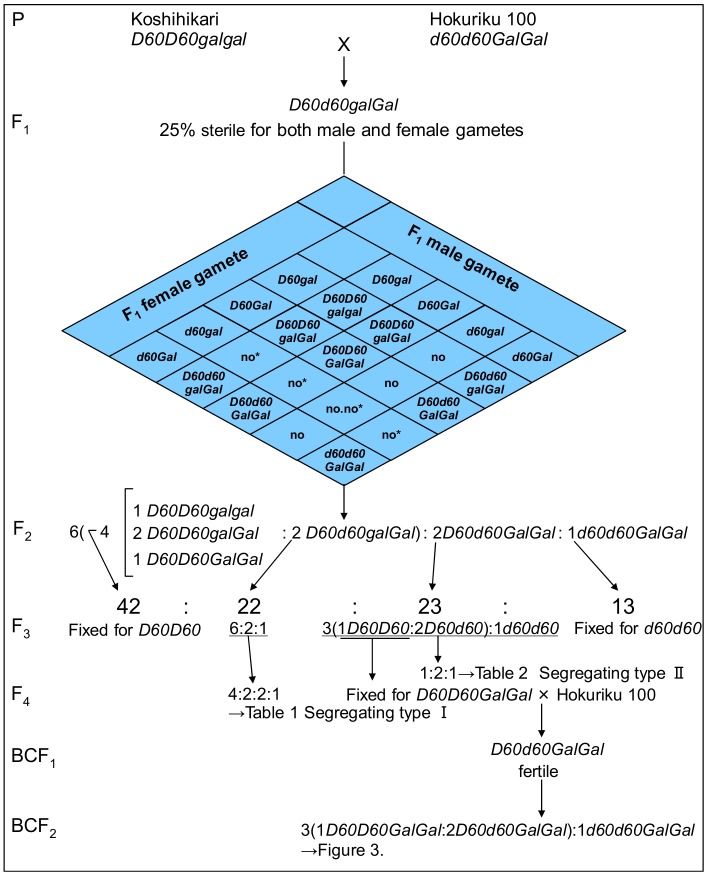
Complementary gamete lethal genetically confirmed through the generation from F_1_ to F_4_ and backcross with *D60Gal* homozygous line. The semidwarfing allele and tall allele were designated as *d60* and *D60*, respectively, and the gametic lethal gene *gal* (activated by *d60*) in Koshihikari, and that the induced opposite allele *Gal*, a gametic non-lethal allele, in Hokuriku 100 were hypothesized. This hypothesis enables the F_2_ progenies of Koshihikari (*D60D60galgal*) × Hokuriku 100 (*d60d60GalGal*) to segregate into the ratio of 1 semidwarf (1 *d60d60GalGal*):2 tall and quarter sterile (2 *D60d60Galgal*):6 tall (2 *D60d60GalGal*:1 *D60D60GalGal*:2 *D60D60Galgal*:1 *D60D60galgal*), because of the gamete lethality of both male and female gametes carrying *gal* and *d60*.

F_1_ to F_3_ of Koshihikari × Hokuriku 100 were retested in this study. Then, a progeny test was carried out on 100 F_3_ lines (30 plants per line) raised from randomly selected F_2_ plants. The F_3_ lines were grouped into four classes as shown in Figure 1, where the author identified two types of segregation lines: segregation type I and segregation type II. Segregation type I was composed of 22 F_3_ lines, derived from partially sterile long-culm F_2_ plants, and was observed to segregate both for culm length and seed fertility, as for F_2_ segregation. Four F_3_ lines (25 plants/line) were selected from these 22 segregation type I lines, and the seed set percentage of each F_3_ plant was counted. Then, 100 F_4_ lines (30 plants/line) were raised from each of the F_3_ plants. Segregation type II lines were composed of 22 F_3_ lines, derived from fertile long-culm F_2_ plants, and were observed to segregate for culm length, but not seed fertility. 100 F_4_ lines (30 plants/line) were raised from each plant of 4 F_3_ lines (25 plants/line) selected from 23 segregation type II lines. The F_4_ plants were investigated for culm length, seed fertility, and days to heading.

Six F_4_ plants were randomly selected from long fixated F_4_ lines, genotype *D60D60GalGal*, in segregation type II, and were backcrossed with Hokuriku 100. Six BCF_1_ plants and 248 BCF_2_ plants were investigated for culm length, seed fertility, and days to heading. 

Plants used in this study were planted 10 cm apart with 30 cm between rows in the experimental field of the Faculty of Agriculture, Tottori University, Tottori, Japan.

### 2.2. Genetic Mapping

In order to determine the chromosomal locations of the gametocidal gene *gal*, we conducted genetic linkage analyses of *gal* on the basis that the segregation ratios of the marker genes linked to *gal* do not fit the Mendelian ratio of 3:1. For the analyses, we developed F_2_ hybrids of the Koshihikari d60Gal line (Koshihikari*7//Koshihikari/Hokuriku 100) and 23 marker gene lineages, which were selected such that they cover all rice chromosomes, taking into account the expectation that the segregation ratios for the marker genes linked to them in the F_2_ differ from the Mendelian ratio of 3:1; in other words, when a recessive marker gene is fully linked to *gal*, this ratio will be 8:1. The Koshihiakri d60Gal line is a isogenic Koshihiakri having *d60* and *Gal*, which was developed by seven times of continuous backcrossing with a recurrent parent Koshihikari and a non-recurrent parent of the *d60* homozygous segregant in the F_2_ of Koshihikari × Hokuriku100.

A chromosome segment substitution line KF2-11-75 (*D60D60galgal*) that carries a segment of Kasalath chromosome 5 in the Koshihikari background was crossed with the Koshihikari d60Gal line (*d60d60GalGal*), and homozygous plants for *d60* (n = 202) were selected from the progenies grown from F_2_ seeds (n = 1854). Then, the *d60Gal* homozygous plants were planted at the Field Science Center. DNA was extracted from the leaves of each short-culm plant, and tested for recombination ratios of *Gal* with simple sequence repeat (SSR) markers that were polymorphic between Kasalath and Koshihikari. Thirty-six SSR markers on chromosome 5 were used to delimit the chromosomal regions bearing *Gal*.

### 2.3. NGS Analysis

The semidwarfing gene *d60* was transferred into Koshihikari by consecutive backcrosses to prepare a semidwarf Koshihikari named Koshihikari d60Gal line. Whole-genome analysis was conducted using the Koshihikari d60Gal line and Koshihikari (*D60gal*). Genomic DNAs were extracted from each cultivar using the hexadecyltrimethylammonium bromide (CTAB) method. Genomic DNA was tagged and fragmented to average 500-bp long using Nextera® transposome. After purification of the transposome using DNA Clean and Concentrator^TM^-5 (Zymo Research, Irvine, CA, USA), adaptors for fixation on the flow cell were synthesized at both ends of each fragment using polymerase chain reaction (PCR). Then, the DNA fragments were subjected to size selection using AMPure XP magnetic beads (Beckman Coulter, Brea, CA, USA). Finally, qualitative and quantitative measurements using a Fragment Analyzer^TM^ (Advanced Analytical Technologies) and Qubit^®^ 2.0 Fluorometer (Life Technologies; Thermo Fisher Scientific, Inc., Waltham, MA, USA) were performed to prepare a DNA library for NGS. The resulting sequenced reads were mapped with BWA software using the Nipponbare genome as a reference, followed by the detection of Single Nucleotide Polymorphisms (SNPs) and Indels using SamTools software.

### 2.4. Pollen Fertility

The fertility of male gametes was examined using 10 F_1_ plants and 40 F_2_ plants (*D60d60Galgal*) following the cross between Koshihikari and Hokuriku 100. Both parents were also examined. Male gamete growth stages were estimated from the auricle length between the flag leaf and the next leaf. Ten panicles were sampled from each plant several times before the meiotic stage (auricle length −10 cm) to the trinucleate pollen stage (+15 cm). Sampled panicles were fixed in formalin-acetic alcohol (FAA) for 48 h and subsequently stored in 70% ethanol. Microspore specimens were prepared by the acetocarmine squash method and observed under a compound microscope. The developmental processes of male gametes were examined by using 10 F_1_ and 40 F_2_ plants. The classification by Kihara and Hirayoshi [24] was adopted for the pollen development process. The diameters of 250 pollen grains per glume were measured at the trinucleate stage with an eyepiece micrometer at 1000 × magnification. The percentage of spikelet fertility was calculated on the basis of the number of filled and unfilled spikelets for each harvested panicle. 

## 3. Results

### 3.1. Genotyping of d60 and Gal Loci through F_1_ to F_4_

F_1_ plants of Koshihikari (tall) × Hokuriku 100 (semidwarf) showed tall phenotypes similar to Koshihikari, but averaged 27.6% unfilled spikelets. F_2_ progenies showed a bi-modal curve with regard to culm length distribution, and were phenotypically classified into 32 semidwarf plants with erect leaves and 278 tall plants. However, this segregation ratio significantly deviated from the 1 semidwarf:3 tall ratio expected from a single recessive gene segregation. In addition to this skewed segregation, the tall F_2_ plants included 71 partially spikelet sterile plants, similar to F_1_. Therefore, the F_2_ population was comprised of three phenotypes; tall and fertile such as Koshihikari, tall and a quarter sterile such as F_1_, and semidwarf and fertile such as Hokuriku 100. The first author speculated that the quarter sterility might be important in revealing the skewed segregation of Hokuriku 100 semidwarfness, so 100 randomly selected F_2_ plants were also used to carry out a progeny test of F_3_ (30 plants per line) in this study.

Four phenotypic classes were observed in the F_3_ population, as shown in Figure 1: 13 F_3_ lines derived from semidwarf F_2_ plants were uniform for semidwarfness and normal fertility; 22 F_3_ lines derived from quarter sterile F_2_ plants were segregated into tall plants, tall and quarter sterile plants, and semidwarf plants as for F_2_ (segregation type I); 65 F_3_ lines derived from tall and fertile F_2_ plants were classified as either uniform for tallness and normal fertility (42 lines) or segregated into semidwarf plants and tall plants in accordance with a Mendelian 1:3 ratio (23 lines, segregation type Ⅱ). These data were almost the same as previous data [21,22] and raised the hypothesis that the semidwarfness of Hokuriku 100 is controlled by a single recessive gene, and that the quarter sterility of tall parents results in the observed skewed segregation of semidwarfness to less than 25% of the next generation. Namely, the semidwarfing allele and tall allele were designated as *d60* and *D60*, respectively, and the first author hypothesized that the gametic lethal gene *gal* (activated by *d60*) must be present in Koshihikari, and that the induced opposite allele *Gal*, a gametic non-lethal allele, must be present in Hokuriku 100. As Figure 1 indicates, this hypothesis enables the F_2_ progenies of Koshihikari (*D60D60galgal*) × Hokuriku 100 (*d60d60GalGal*) to segregate into the ratio of 1 semidwarf (1 *d60d60GalGal*):2 tall and quarter sterile (2 *D60d60Galgal*):6 tall (2 *D60d60GalGal*:1 *D60D60GalGal*:2 *D60D60Galgal*:1 *D60D60galgal*), because of the gametic lethality of both male and female gametes carrying *gal* and *d60*. The observed segregation ratio of 13:22:23:42 in the F_3_ classification represents a good fit to the theoretical ratio of 1 *d60d60GalGal*:2 *D60d60Galgal*:2 *D60d60GalGal*:4 (1 *D60D60GalGal*:2 *D60D60Galgal*:1 *D60D60galgal*) (χ^2^ = 0.49, 0.90 < *p* < 0.95) based on the above hypothesis.

**Figure 2:**
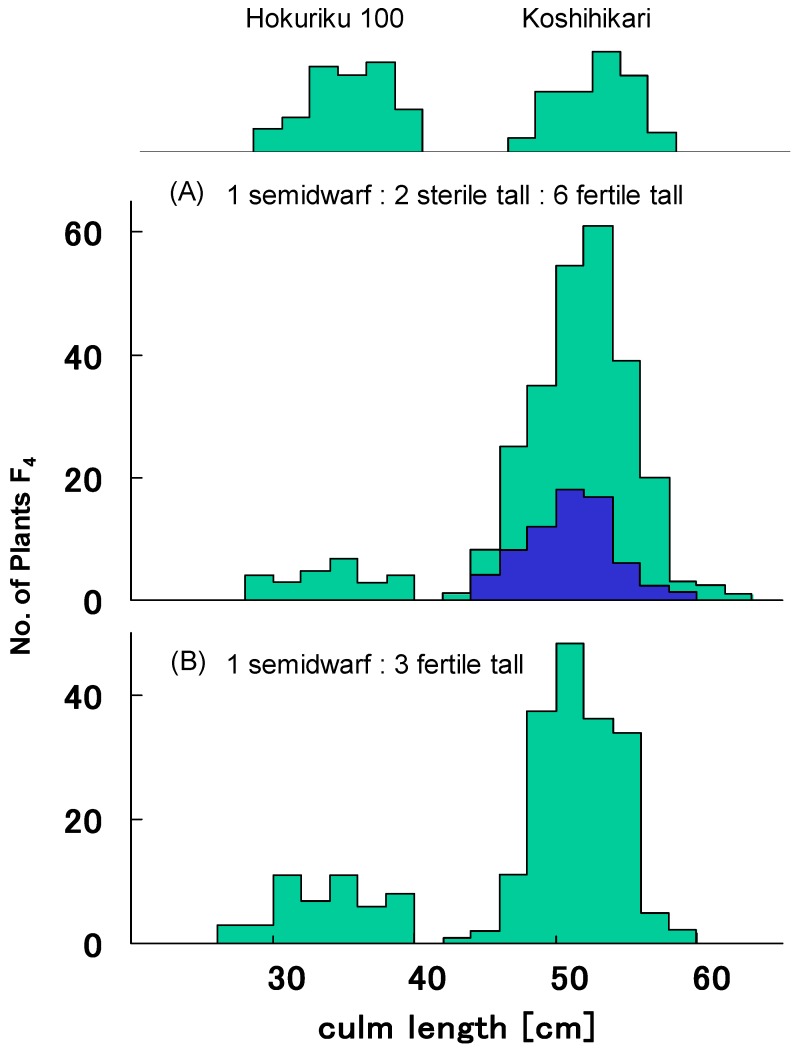
Frequency distribution of culm length in pooled F_4_ progenies derived from segregation type I F_3_. (**A**) Pooled F_4_ progenies of sterile 24 F_3_ plants were segregated into semidwarf plants (n = 61), partially sterile long plants (n = 114), and fertile long plants (406), which showed a good fit to the 1:2:6 ratio expected from the existence of *gal* (χ^2^ = 2.86, 0.20 < *p* < 0.30). (**B**) Pooled F_4_ progenies of fertile 19 F_3_ plants were segregated into semidwarf plants (122) and long plants (356), showing a good fit to single recessive gene segregation ratio 1:3 (χ^2^ = 0.07, 0.90 < *p* < 0.95).

Table 1 shows representative distributions of culm length and seed fertility in 100 F_4_ lines from segregation type I. F_4_ lines were classified to four classes, as for F_3_, on the basis of frequency distribution for culm length and seed fertility. All plants from 11 F_4_ lines derived from semidwarf F_3_ plants with an average 93.6% seed set percentage showed semidwarfness and normal seed fertility with over 90% seed set percentage. Twenty-four F_4_ lines from partially sterile long F_3_ plants with an average seed set percentage of 70.9% were segregated into semidwarf plants (n = 61), partially sterile long plants (n = 114), and fertile long plants (406). Plants pooled from these 24 F_4_ lines showed a good fit to the 1:2:6 ratio expected from the existence of *gal* (χ^2^ = 2.86, 0.20 < *p* < 0.30) (Figure 2A). On the other hand, 65 F_4_ lines from fertile long F_3_ plants with an average seed set percentage of 94.7% showed normal seed fertility with over 90% seed set percentage. As in Figure 2B, 19 of these 65 lines were segregated into semidwarf plants (122) and long plants (356), showing a good fit to single recessive gene segregation ratio 1:3 (χ^2^ = 0.07, 0.90 < *p* < 0.95). The other 46 F_4_ lines were fixed as long plants. This F_4_ classification showed a good fit to the theoretical ratio 1 *d60d60GalGal*:2 *D60d60Galgal*:2 *D60d60GalGal*:4 *D60D60* homozygous [1*GalGal*:2*Galgal*:1*galgal*], as expected from existence of the *gal*/*Gal* locus (χ^2^ = 0.67, 0.80 < *p* < 0.90).

### 3.2. d60 is Inherited as a Single Recessive Gene in the Non-Gamete Lethal Gal-Homozygous Background

All the F_4_ plants from segregation type II had normal seed fertility of over 90%; so, 100 F_4_ lines were classified according to the frequency distribution for culm length. Table 2 shows the representative frequency distributions for the culm length in several lines of each class. All the F_4_ plants in 24 lines from semidwarf F_3_ plants showed semidwarfism. Forty-nine out of 76 F_4_ lines from long F_3_ plants segregated into a 141 semidwarf:440 long plants ratio, which is a good fit with the theoretical ratio of 1 semidwarf:3 long expected if semidwarfism is controlled by a single recessive gene (χ^2^ = 0.17, 0.50 < *p* < 0.70). The remaining 27 F_4_ lines were all fixed as long plants. This F_4_ ratio of 24:49:27 showed a good fit to the theoretical 1:2:1 ratio expected from a single recessive gene model of semidwarfness (χ^2^ = 0.22, 0.80 < *p* < 0.90).

**Figure 3:**
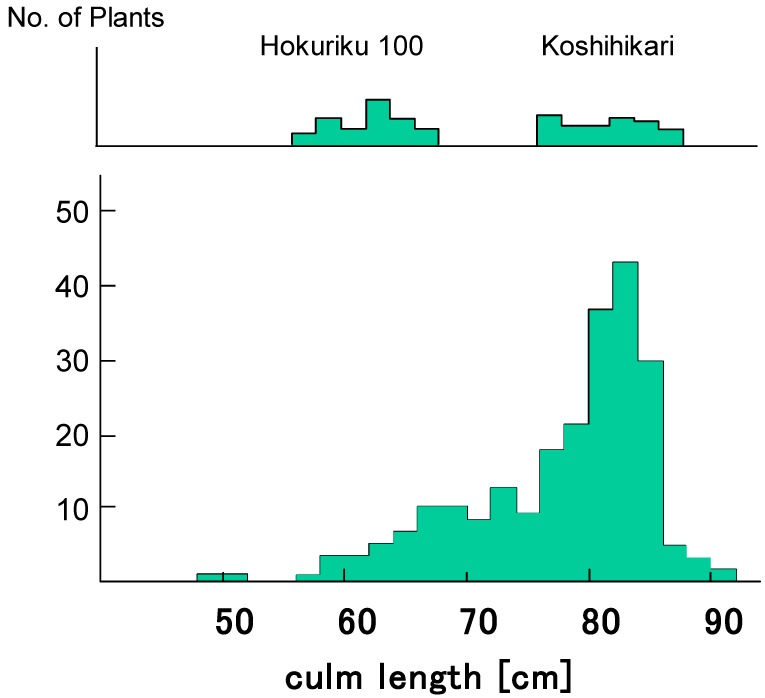
Frequency distribution for culm length in the BCF_2_ progenies of *D60D60GalGal* line × Hokuriku100 (*d60d60GalGal*). BCF_2_ plants segregated as 67 semidwarf:181 long plants, which shows a good fit to the theoretical 1:3 ratio expected from a single recessive gene model (χ^2^ = 0.54, 0.30 < *p* < 0.50). Therefore, *d60* is inherited as a single recessive gene in the *D60d60GalGal* genetic background (i.e., in the absence of *gal*).

**Figure 4:**
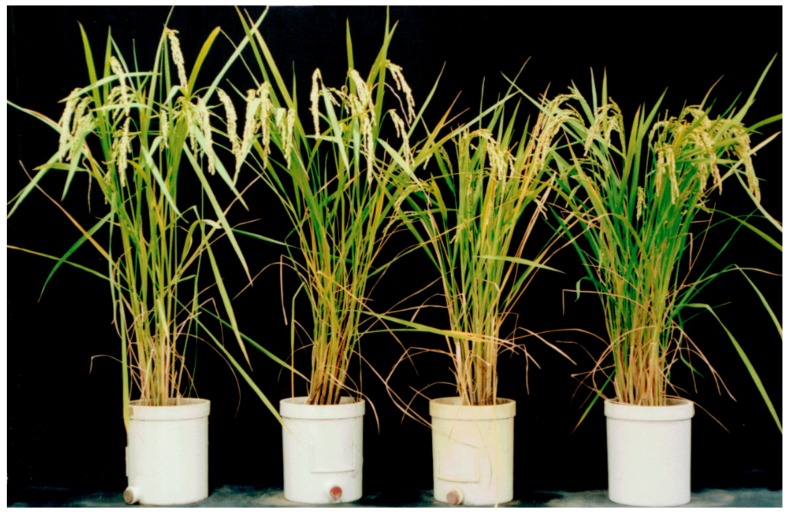
Segregation for plant type in the BCF_2_ progenies of *D60D60GalGal* F_4_ line × Hokuriku100 (*d60d60GalGal*). From left to right: *D60D60GalGal* F_4_ line, Tall BCF_2_, Semidwarf BCF_2_, and Hokuriku100 (*d60d60GalGal*).

Six Koshihikari-type long F_4_ plants were randomly selected from 27 long F_4_ lines, genotype *D60D60GalGal*, in segregation type II and were backcrossed with Hokuriku 100. BCF_1_ plants showed normal fertility with a seed set of 96.0% and a pollen fertility of 97–98%. Figure 3 shows the segregation of BCF_2_ plants as 67 semidwarf:181 long plants (Figure 4), which shows a good fit to the theoretical 1:3 ratio expected from a single recessive gene model (χ^2^ = 0.54, 0.30 < *p* < 0.50). Therefore, *d60* is inherited as a single recessive gene in the *D60d60GalGal* genetic background (i.e., in the absence of *gal*). The plant types of segregants were clearly classified based on the phenotype, as shown in Figure 4.

### 3.3. Genetic Mapping of Gal Loci

**Figure 5:**
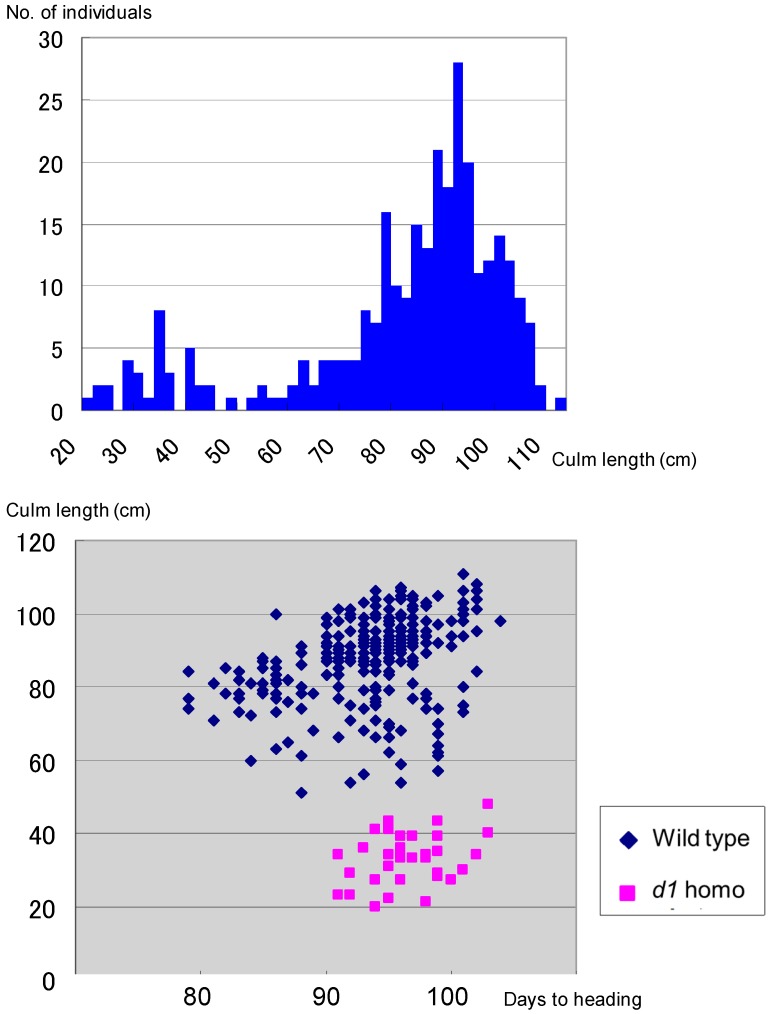
Little segregation of *d1* homozygotes in the F_2_ between the Koshihikari d60Gal line and *d1* line, showing a ratio of 263 wild type:34 *d1* homozygote. It is close to the theoretical segregation ratio of 8:1 at the *d1* locus (χ^2^ = 0.03, 0.80 < *p* < 0.90), when completely linked to the *gal* locus, indicating a genetic linkage between *d1* and *gal* loci on chromosome 5.

**Figure 6:**
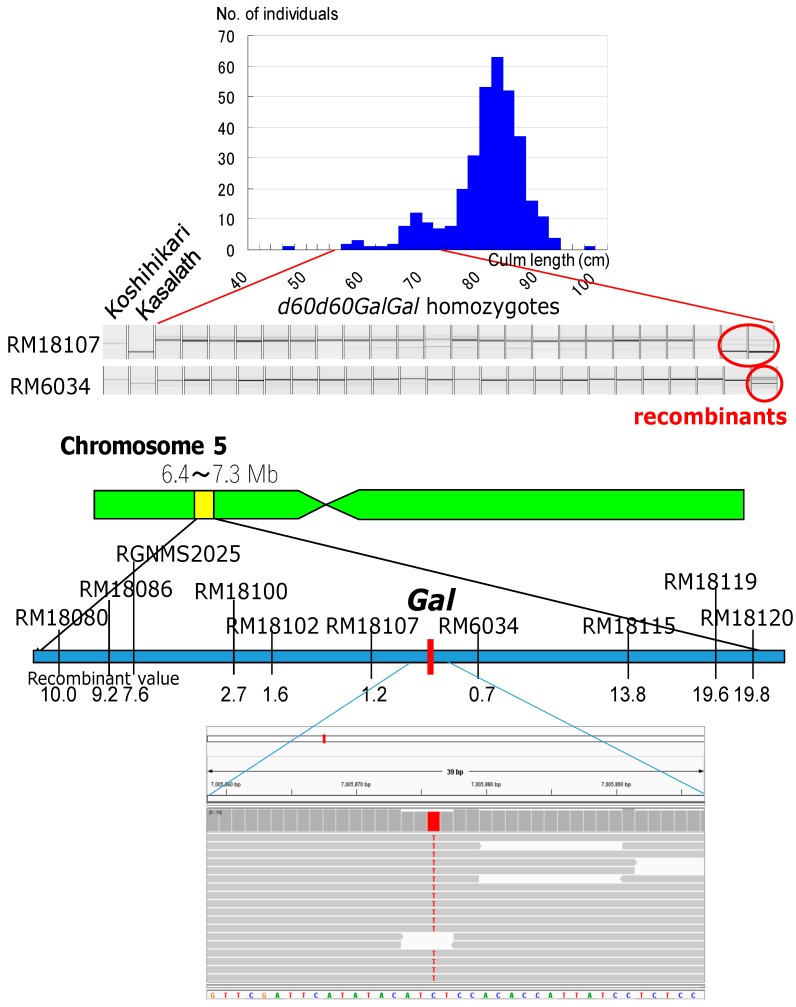
Molecular mapping of gamete lethal gene *Gal* and identification of candidate single nucleotide polymorphism (SNP) responsible for *Gal* by whole genome analysis using Next generation sequencer. Koshihikari d60Gal line was crossed with chromosome segment substitution line that carry segment of chromosome 5 of the *indica* cultivar ‘Kasalath’ in the background of the *japonica* cultivar ‘Koshihikari’. Short-culm homozygous (*d60d60GalGal*) plants in the F_2_ progenies were examined for genetic linkage by using SSR markers located on chromosomes 5. Three markers—namely, RM18102, RM18107, and RM6034—in the region 7.0 Mb away from the distal end, were liked with *Gal* with recombination values of 1.6, 1.2, and 0.7, respectively. These results indicate that the *Gal* locus is located around 7.0 Mb away from the distal end of the short arm of chromosome 5. We found a SNP from C to T in Koshihikari d60Gal by Nextgen sequencing, which was located at 7,005,876 bp from the end of the short arm of chromosome 5 at the center between RM18107 and RM6034. It is highly possible that the SNP at 7,005,876 bp is responsible for the mutation of *Gal*.

Genetic linkage analysis of the F_2_ progenies of the cross between the Koshihikari d60Gal line (*d60d60GalGal*) and a line carrying a gene marker *d1* on chromosome 5 showed that the segregation ratio of wild type to *d1* homozygote was 263:34 (Figure 5). This is a marked distortion from the Mendelian segregation ratio, but it is close to the theoretical segregation ratio of 8:1 at the *d1* locus (χ^2^ = 0.03, 0.80 < *p* < 0.90), when completely linked to the *gal* locus, indicating a genetic linkage between *d1* and *gal* loci on chromosome 5. Next, the Koshihikari d60Gal line was crossed with chromosome segment substitution lines that carry segments of chromosome 5 of the *indica* cultivar ‘Kasalath’ in the background of the *japonica* cultivar ‘Koshihikari’. Short-culm homozygous (*d60d60GalGal*) plants in the resulting F_2_ progenies (Figure 6) were examined for genetic linkage by using SSR markers located on chromosomes 5, thereby achieving fine mapping of the *Gal* loci. Three markers—namely, RM18102, RM18107, and RM6034—in the region 7.0 Mb away from the distal end, were liked with *Gal* with recombination values of 1.6, 1.2, and 0.7, respectively (Figure 6). These results indicate that the *Gal* locus is located around 7.0 Mb away from the distal end of the short arm of chromosome 5.

### 3.4. Identification of Gal Responsible SNP by NGS Analysis

Using next generation sequencer, we obtained a total read number of 66,155,260 with an average length of 124 bp in Koshihikari and a total read number of 126,884,326 with an average length of 125 bp in Koshihikari d60Gal. By mapping 99.91% of the reads of Koshihikari using the Nipponbare genome sequence as the reference, we attained the consensus sequence of Koshihikari with a total length of 372,912,445 bp bearing a mean coverage of 12.79. Then, 99.88% of reads of Koshihikari d60Gal were mapped using the consensus sequence of Koshihikari as the reference. The mean coverage was 22.42. Furthermore, we prepared vcf files of entire genomes and compared the whole-genome sequences of Koshihikari d60Gal with the virtual Koshihikari genome. As a result, we found a SNP from C to T in Koshihikari d60Gal, which was located at 7,005,876 bp from the end of the short arm of chromosome 5 in the Koshihikari genome (Figure 6). This SNP was situated almost at the center between the nearest SSR markers, RM18107 and RM6034, which were both linked with *Gal*. To survey DNA mutations over the 6–8 Mb region of Chromosome 5, there were no sequence alterations excerpt for this SNP, between the Koshihikari (*d60gal*) and Koshihikari d60Gal. We conducted a high coverage of Nextgen sequencing, so the SNP was certainly specific to the region of Koshihikari d60Gal chromosome 5. Therefore, it is highly possible that the SNP at 7,005,876 bp is responsible for the mutation of *Gal*. In this region, there were no annotated sequences in the rice annotation project database (https://rapdb.dna.affrc.go.jp/). However, the region surrounding the SNP showed homologies to some hypothetical proteins of humans or swallowtails. Genetically, the role of the *Gal* allele is to transmit *d60* in viable gametes, whereas that of *gal* is to reduce the transmission by complementary gamete death. Functional analysis for such unknown proteins would be a future issue to research.

### 3.5. Coexistence of d60 and Gal Lose Vegetative Nuclei but Two Generative Nuclei

**Figure 7:**
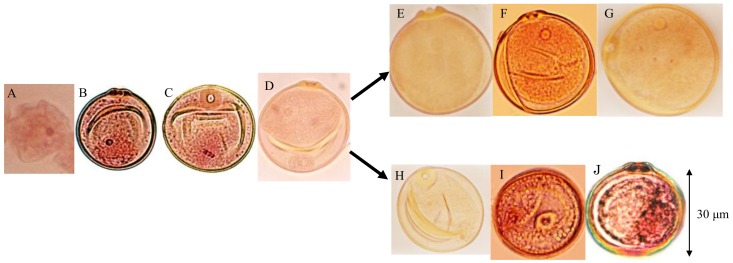
Developmental process of male gametes in F_1_ and 25% sterile F_2_ plants (genotype *D60d60Galgal*). (**A**) First shrunk pollen, (**B**) single-nucleate pollen, (**C**) metaphase of first-pollen mitosis, (**D**) early binucleate pollen, (**E**) late binucleate pollen, (**F**) second pollen mitosis in late binucleate pollen, (**G**) mature trinucleate pollen before flowering, (**H**) abortive binucleate pollen (genotype *d60gal*), (**I**) second pollen mitosis in abortive pollen losing vegetative nuclei (*d60gal*), (**J**) remnant of two generative cells in degraded pollen before flowering (*d60gal*).

Pollen fertility was examined using panicles sampled before anthesis from both parents, 10 F_1_ plants (*D60d60Galgal*) and 40 randomly chosen F_2_ plants. Eight out of 40 F_2_ plants showed partial seed fertility varying from 69.2–73.8% (average, 71.9%), and the remaining 32 F_2_ plants showed a normal seed set varying from 95.3–97.8% (average, 96.7%) in maturity. Gamete development was observed. Meiosis were normally observed in all plants, which was the same as tentative data using F_4_ partial sterile plants [25]. After releasing from the tetrads, microspores became the first stranded stage (Figure 7A). At the single nucleate pollen stage, wall and germ pores were formed, and pollens became vacuolated (Figure 7B). During the first pollen mitosis, metaphase chromosomes were visible, and cytoplasm developed (Figure 7C). Binucleate pollens having both generative and vegetative nuclei were normally observed in all plants (Figure 7D). At the early binucleate stage, generative nuclei became enclosed in newly formed generative cells and were located opposite the pore side, apart from the vegetative nuclei (Figure 7D). However, some of the pollens discontinued development in the binucleate stage, and their vegetative nuclei became smaller in the F_1_ and 25% seed-sterile F_2_ plants (Figure 7H). On the contrary, in the other normal pollens, generative nuclei again approached the vegetative nuclei in the late binucleate stage (Figure 7E) and were divided into two generative nuclei by the second-pollen mitosis (Figure 7F) and finally developed into normal trinucleiate pollens (Figure 7G). On the other hand, in the abortive pollens vegetative nuclei are losing, but second pollen mitosis was observed (Figure 7I), and remnant of two generative cells were observed in degraded pollens before flowering (Figure 7J).

**Figure 8:**
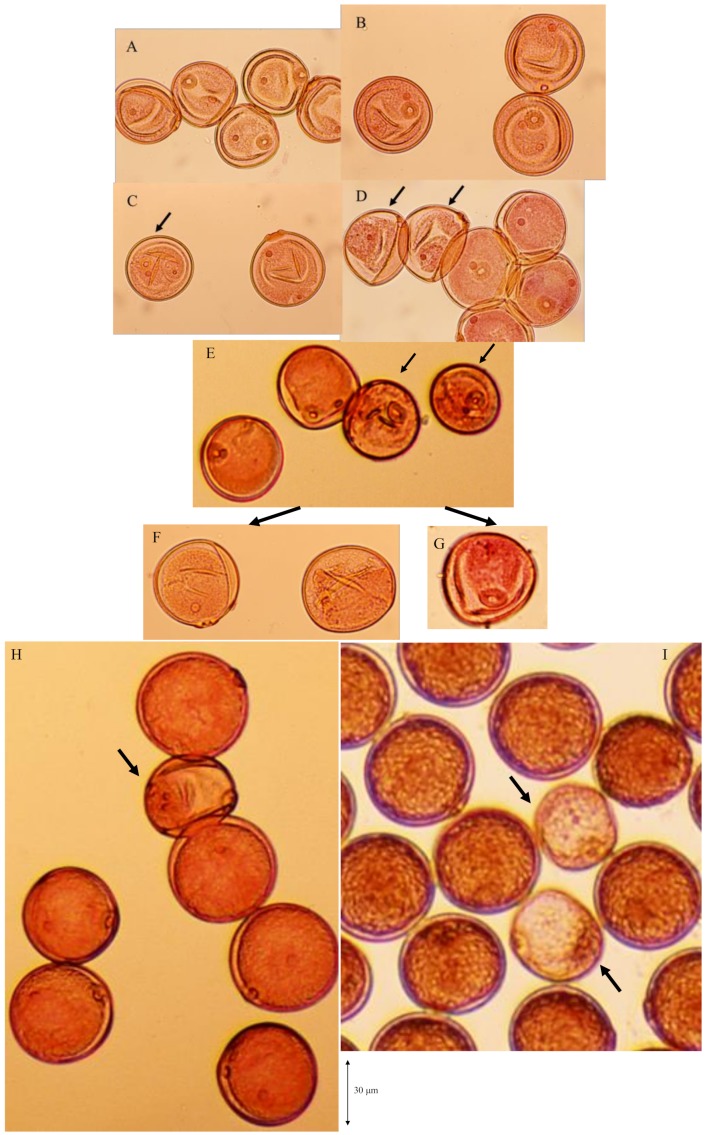
Massive observation of degradation process of male gametes in 25% sterile plants (genotype *D60d60Galgal*). (**A**) single nucleate stage, (**B**) early binucleate pollen stage, (**C**) degradation of generative cell in some early binucleate pollen (arrow), (**D**) degraded pollen losing vegetative nuclei and holding only a generative nuclei (arrows) at the late binucleate pollen stage, (**E**) degraded pollen at the late binucleate pollen stage (arrows), (**F**) metaphase of second pollen mitosis in normal pollen, (**G**) metaphase of second pollen mitosis in abortive pollen, (**H**),(**I**) abortive pollen holding only two generative nuclei (arrows) among mature pollens before flowering.

**Figure 9:**
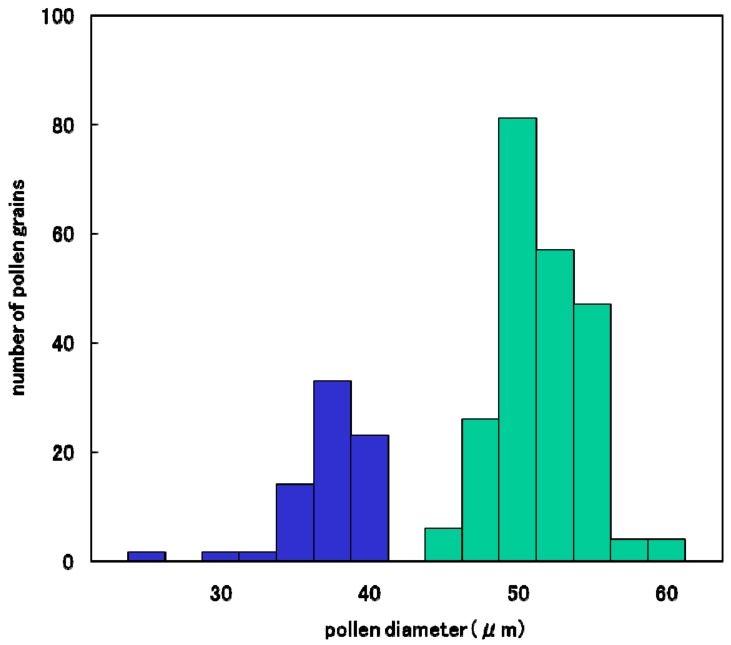
Frequency distribution of pollen diameter in a partially sterile F_2_ plant (*D60d60Galgal*) derived from Koshihikari × Hokuriku 100. 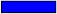: Empty pollen.

The degradation process of male gametes in 25% sterile plants (genotype *D60d60Galgal*) are massively shown in Figure 8. The single nucleate stage is normal (Figure 8A) and enter the early binucleate stage (Figure 8B). However, the degradation of generative cell started in some binucleate pollen (Figure 8C). Degraded pollens lost vegetative nuclei and contain only a generative nuclei (Figure 8D,E) at the late binucleate stage. Second pollen mitosis is observed in normal pollens (Figure 8F) and degraded pollens, which lost vegetative nuclei (Figure 8G). Degraded pollens holding only two generative nuclei were observed among mature pollens, and finally became almost empty before flowering (Figure 8H,I). As a result, two distinguishable types of pollen were observed before anthesis in F_1_ and 25% seed-sterile F_2_ plants; degenerated vacant pollens with only a remnant of generative cell and small diameter around the median value of 36 microns (Figure 7J, Figure 8H,I), as well as normal trinucleate pollens with well-developed cytoplasm and normal diameter around the median value of 52 microns (Figure 7G, Figure 8H, I). Figure 9 shows the frequency distribution for pollen diameters in the glume of a partially seed sterile F_2_ plant, in which it is possible to distinguish between pollen types according to diameter. Vacant pollen diameters were distributed around a median value of 36 µm, while normal pollens were distributed around a median value of 52 µm.

**Figure 10:**
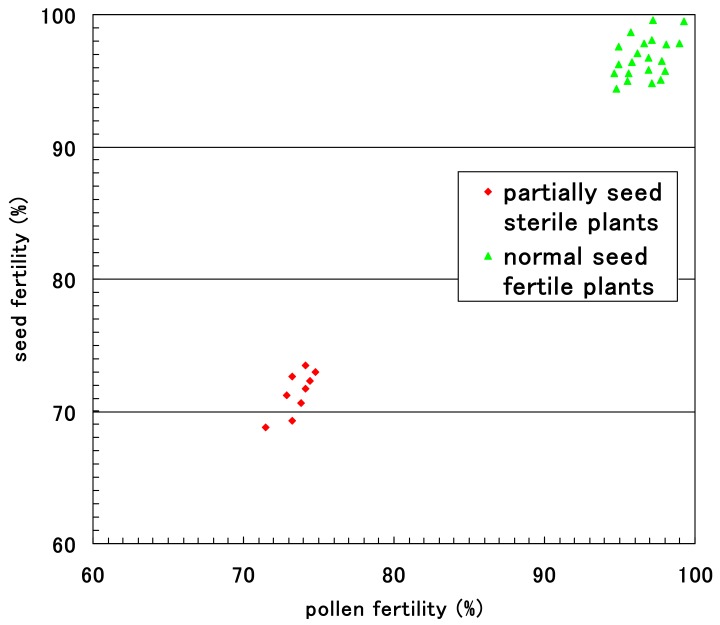
Relationship between pollen fertility and seed fertility in the F_2_ progenies of partially sterile F_1_ plants (*D60d60Galgal*) of Koshihikari × Hokuriku 100.

The pollen fertility of each F_2_ plant was obtained as the rate of normal pollen with a large diameter and stainable cytoplasm. Small, empty pollen (average, 25.3%) was observed together with stainable mature pollen in all F_1_ plants and eight F_2_ plants with partial seed setting at maturity. These partially seed sterile F_2_ plants had an average of 71.9% seed fertility and an average of 74.7% pollen fertility. Fewer degraded pollen grains were observed in the 32 F_2_ plants with nearly complete seed setting, resulting in a pollen fertility of 99.4%. Figure 10 shows the relationship between pollen fertility and seed fertility in 40 F_2_ plants. Only partial seed sterile plants showed partial pollen sterility. 

The average lethal rate of pollen in partially seed sterile F_2_ plants was calculated from the reduced rate of normal pollen from normal seed fertile F_2_ plants using the Equation (1):

(99.4% − 74.7%/99.4%) × 100(1)

The small, empty pollen averaged 24.8%, which is in agreement with the theoretical expected frequency of the haploid genotype *d60gal* in eight F_2_ plants with a 71.9% seed set. As 75.4% of normal pollen in all F_1_ plants and eight F_2_ plants is fertile, the observed 27.6% unfilled spikelets must be caused by infertility of the embryo sac. 

Female fertility was determined as the seed fertility. The average lethal rate of female gametes in partially seed sterile F_2_ plants was calculated as 25.6% from the reduced rate of normal ovules from normal seed fertile F_2_ plants using the Equation (2):

(96.7% − 71.9%/96.7%) × 100(2)

The lethal rates of male and female gametes (24.8% and 25.6%, respectively) coincide with the theoretical 25% lethality of male and female gametes from the coexistence of both *d60* and *gal* and indicates the existence of the gametic lethal gene *gal*. Consequently, a quarter of both sex gametes were aborted in the F_1_ plants and some of the tall F_2_ plants of Koshihikari × Hokuriku 100 (*D60d60Galgal*).

## 4. Discussion

The gametic lethal gene *gal* was identified in the present study, together with its activator *d60* (semidwarfing gene), in a cross between semidwarf mutant Hokuriku 100 and its original tall variety Koshihikari. The F_2_ progeny from these F_1_ hybrids displayed a unique heredity style of segregating into the ratio of 6 fertilizable long culms (4*D60D60*:2*D60d60GalGal*):2 partially non-fertilizable long culms (*D60d60Galgal* = F_1_ type):1 semidwarf (*d60d60GalGal*), which deviated from the Mendelian 3:1 ratio. The appearance of partial seed sterility in F_1_, and F_1_-type partial seed sterility in many long-culm F_2_ plants also assisted in identification of *gal* and *d60*.

Male gametes carrying *gal* and *d60* develop into lethal pollen, such that *d60* is not transmitted to progeny without *Gal*. In other words, *Gal* is indispensable to the heredity of *d60*. The dwarf gene *d60* could not have been originally obtained without the accidental simultaneous mutation of two genes, *gal*→*Gal* and *D60*→*d60*. The hybrid sterile genes *gal* and *d60* identified from crosses between closely related *japonica* varieties differ from most known hybrid sterile genes identified from crosses between distantly related species belonging to different gene pools with reproductive barriers. This was the first discovery of a hybrid sterility gene among *japonica* varieties free from a reproductive barrier. 

Hybrid sterility is often found among distantly related taxa of plants and animals. In rice cultivars (*Oryza sativa* L.), F_1_ hybrids between the two major subspecies, *indica* and *japonica*, usually show partial sterility of gametes [26,27,28,29]. This involves several genetic systems such as pollen sterility by the duplicate gametophytic system by recessive *s* alleles on the two *S* loci [30,31], female sterility caused by one-locus sporo-gametophytic allelic interaction by the single *S* locus [32,33,34,35,36,37,38,39], and both-sex breakdown according to the one-locus gene model [40]. This hybrid sterility from *indica/japonica* crosses causes serious problems in developing F_1_ varieties or breeding programs utilizing these divergent germplasms. 

Oka [30] proposed that duplicate *S* gene loci, which work as developmental factors in gametes, cause hybrid sterility when the F_1_ gametes receive both recessive *s* genes on each duplicate locus. For example, if parents A and B have genotypes *s1/s1 +2/+2* and *+1/+1s2/s2*, respectively, in which at least one + gene is necessary for normal development of the gamete, then 25% of their F_1_ hybrids will be sterile. This is because those gametes carrying the double recessive combination *s1s2* deteriorate due to deficiencies during gamete development. These hybrid sterility is similar to that caused by *gal* and *d60* in that two genes are responsible for both systems. However, *gal* and *d60* cause both sex sterilities, whereas Oka [31] suggests that the duplicate *s* gene model can only explain male gamete sterility.

Kitamura [32] explained female sterility in *indica/japonica* hybrids by the one locus sporo–gametophytic interaction hypothesis—that is, disharmony between one allele in the gamete and another in the surrounding sporophytic tissues. This model assumes parent genotypes of *S/S* and *S_a_/S_a_* creating the hybrid *S/S_a_*, in which allele *S* present in the maternal tissue induces an abortion of gametes carrying the opposite allele, *Sa*. Thus, 50% of *S/Sa* plants are sterile and produce gametes carrying the *S* allele only; selfed progenies are all fertile. Ikehashi [41] showed that this one locus model was a more likely explanation for *indica/japonica* hybrid sterility than the two loci model [30,31]. The allelic interaction model [35] has been accepted as the genetic basis of hybrid sterility. According to the model, most of the sterility in F_1_ hybrids is caused by an allelic interaction in the heterozygote of the *S_5_^i^* allele and *S_5_^j^* allele at the *S_5_* locus, where *indica* and *japonica* varieties have *S_5_^i^* and *S_5_^j^* alleles, respectively. The *indica*/*japonica* heterozygotes (*S_5_^i^*/*S_5_^j^*) genotype is semisterile due to the partial abortion of female gametes carrying the *S_5_^j^* allele. On the other hand, some *javanica* rice varieties carry the neutral allele *S_5_^n^*, and genotypes *S_5_^n^/S_5_^i^* and *S_5_^n^/S_5_^j^* do not show hybrid sterility. The donor of *S_5_^n^* is referred to as a wide compatible variety (WCV) [35], and this allele has been incorporated into *indica* and *japonica* varieties to overcome sterility barriers in hybrid rice breeding [42,43]. The chromosomal location of *S_5_^j^* has been analyzed by using restriction fragment length polymorphism (RFLP) markers [44]. Thus, Qiu et al. [45] were able to delimit *S_5_* to a 40-kb DNA fragment on chromosome 6, by constructing a population from a three-way cross based on near-isogenic lines (NILs) for the *S_5_* locus. Finally, the *S_5_* locus has been successfully cloned [46].

In the subsequent studies based on analyses of the fertility of a number of *indica* × *japonica* hybrids, over 30 female gametes’ sterility loci, including major genes and quantitative trait loci (QTLs), were identified and mapped [47,48,49,50,51,52,53,54,55], or male gametes’ sterility have been identified [53,56,57,58,59,60,61]. So far, indica/japonica hybrid sterility loci were identified on chromosomes 4, 6, 7, 12, and 1, which lead to female gamete abortion through allelic interactions: *S_7_* [47], *S_8_* [48], *S_9_*, and *S_15_* [39] and *S_16_* [49], etc. Among them, the *Sa* locus has been successfully cloned [62]. One-locus allelic interactions for male sterility were also recognized in hybrids between two cultivated rice species *O. sativa* and *O. glaberrima* Steud. [63,64,65], *O. sativa* and *O. rufipogon* [66], and *O. sativa* and *O. glumaepatula* [67] and series of *S_1_* [65,68], *S_18_* [68], *S_20_*, *S_21_* [63,64], *S_22A_*, and *S_22B_* were identified [67]. Above all, hybrid sterilities in rice can be explained by a single locus allelic interaction. Therefore, hybrid sterility caused by the two genes *d60* and *gal* is an extremely rare case in rice. Moreover, gamete breakdowns of both sexes, as for *gal* and *d60*, are particularly rare, with the exception of *S_10_*, which caused a one-locus allelic interaction [69].

On the other hand, the monogenetic male-sterile gene including the photoperiod-sensitive male sterile (PGMS) and thermosensitive male sterile are useful to facilitate the production of F_1_ seeds [70] or the intercrossing phase of recurrent selection. Several genes for PGMS and thermosensitive male sterile were mapped or isolated [71,72,73]. However, their monogenetic inheritance and the expression of male sterility are certainly distinguished from the complementary sterility caused by the two genes *d60* and *gal.*

For other plant species, generally, genic models of hybrid sterility by sporo–gametophytic allelic interaction at a single locus have been proposed as gamete eliminators, which cause the abortion of gametes due to allelic interaction, and were first reported in tomato plants by Rick [74] and have since been shown to be widely distributed in interspecific plant hybrids [75]. Gametic selection in tomato hybrids is caused by the gamete eliminator *Gep*, which induces the abortion of both male and female gametes carrying the opposite allele in the heterozygote *Gep*/*Gec* [74] and the pollen killer locus [76]. In addition, the preferential transmission of alien chromosomes common to interspecific and intergeneric hybrids of *Nicotiana* and wheat are explained by assuming that a similar sterility factor(s) to gamete eliminator or sporo–gametophytic interaction is located on the alien chromosome [77,78,79,80]. In the case of the pollen killer locus, an alien chromosome introduced into *Triticum aestivum* from *Aegilops triuncialis* caused an inviability of gametes lacking this chromosome, resulting in the preferential transmission of the *Aegilops* chromosome to the offspring. A similar case of sporo–gametophytic interaction was also found between *T. aestivum* and *Ae. longissima* or *Ae. sharonensis* [78,81]. Above all, gametocidal genes or chromosomal fragments causing an abortion of gametes have been reported for many plant species. Accumulated evidence suggests that the phenomenon of gamete abortion through allelic interaction is widespread between distantly related taxa, serving as one of the genetic mechanisms for reproductive barriers [64,65,82]. Therefore, hybrid sterility caused by the two genes *d60* and *gal* is an extremely rare case in the plant kingdom.

The abnormal segregation of semidwarfness in the present study aided the discovery of the gametic lethal model composed of *d60* and *gal*. The abnormal segregation of some marker genes has been explained by their linkage to gametophytogenes, which control the fertilization ability of pollen. Rice has 10 gametophytogenes that are designated *ga-1* to *ga-10*, some of which have been mapped onto four loci [83,84,85,86,87,88,89]. Although many genes were reported from varietal crosses within *O. sativa* [85,86,87,90], these gametophytogenes did not cause seed sterility. Therefore, it was apparent that *gal* differed from gametophytogenes in this way. In addition, segregation distortion was observed at a number of loci in inter-subspecific hybrids [91,92,93].

If *Gal* had not originally mutated from *gal* together with the induction of *d60* from *D60*, *d60* would have been eliminated by the lethality of M_1_ gametes, and *gal* would not have been identified as a gametic lethal gene. Thus, *d60* and its transmitter *Gal* are rare and valuable mutant genes forwarding semidwarf breeding as an alternative of *sd1*. For the practical use of *d60* in semidwarf breeding programs, line *D60D60GalGal* is a special class of germplasm that is capable of producing fertile hybrids when crossed with Hokuriku 100. The early mutation breeding program to create semidwarf Koshihikari, before the Hokuriku National Agricultural Experiment Station, was unsuccessful. Then, Samoto and Kanai [20] enlarged the scale of mutation breeding using 200,000 M_1_ plants. This led to the selection of a semidwarf line Hokuriku 100 from M_5_ plants derived from 298 short mutants selected from 80,000 M_2_ plants. The appearance rate of short mutants at 0.3% was much lower than the 11.0% observed for wheat [94] and the 5.2% observed for barley [95], which may be a result of gametic lethality by interactions between *gal* and induced dwarf genes. 

Extensive typhoon damage from the lodging of rice has become a serious problem in recent years, and developing new varieties of typhoon-resistant rice through the introduction of semidwarf genes is an urgent task. There are high expectations of ‘Hikarishinseiki’ (Hikari New Century) [96], which is a new lodging-resistant, high-yield, tasty variety developed through the introduction of the semidwarf gene *sd1* to Koshihikari. However, in consideration of the maintenance and expansion of genetic diversity, this gene should not be solely relied upon for the development of semidwarf varieties. Through this study, we identified a new semidwarfing gene *d60*, which shows strong lodging-resistance, and genetically independence from *sd1* [23]. Further research could elucidate the function of *d60* and enable the development of novel semidwarf rice varieties.

## 5. Conclusions

The gametic lethal gene *gal* in combination with the semidwarfing gene *d60* causes complementary gamete lethality in rice. Through F_2_ to F_4_ derived from the cross between *D60gal*-homozygous (tall) and *d60Gal*-homozygous (semidwarf), progenies of F_1_ and partial sterile plants (*D60d60Galgal*) segregated in a ratio of 1 semidwarf (1 *d60d60GalGal*):2 tall and quarter sterile (2 *D60d60Galgal*):6 tall (2 *D60d60GalGal*:1 *D60D60GalGal*:2 *D60D60Galgal*:1 *D60D60galgal*), which is skewed from the Mendelian ratio of 1 semidwarf:3 tall. Through F_3_ to F_4_, progenies of fertile and tall heterozygous plants (*D60d60GalGal*) segregated in the Mendelian ratio of 1[semidwarf (*d60d60GalGal*)]:2[1 semidwarf:3 tall (*D60d60GalGal*)]:1[tall (*D60D60GalGal*)]. The backcrossing of *D60Gal*-homozygous tall F_4_ plants with *d60Gal*-homozygous plants resulted in fertile and tall BCF_1_ (*D60d60GalGal*), and BCF_2_ segregated in 1 semidwarf (*d60d60GalGal*)]:3 tall (2 *D60d60GalGal*:1 *D60D60GalGal*), proving that *d60* is transmitted as a single recessive gene in the *D60d60GalGal* genetic background (i.e., in the absence of gal). Further, *gal* was localized on chromosome 5, which was evident from the deviated 1:8 segregation of linked gene *d1* and molecular fine mapping using SSR markers. Next-generation sequencing identified the candidate SNP responsible for *Gal* located at 7,005,876 bp from the end of the short arm of chromosome 5 in the Koshihikri genome. Pollens genotype *d60gal* began to degrade at the binucleate stage and lost vegetative nuclei. However, it underwent second pollen mitosis, raising two generative nuclei still in a small abortive pollen. Thus, our study describes a novel genetic mode bearing a reproductive barrier. This is the first report on such a complementary lethal gene, whose mutation allows the transmission of a co-induced valuable semidwarfng gene *d60*.

## Figures and Tables

**Table 1:** Classification of F_4_ lines of Segregating type Ⅰ based on segregation of culm length and seed fertility.

Phenotype and Genotype of F_3_		Frequency Distribution^1)^ of Culm Length and Seed Fertility in Representative F_4_ Lines																			F_4_ Lines		
		Culm Length (cm)																			Observed No.	Expected	
			50					60					70					80				No.	Ratio
Hokuriku 100 type (semidwarf)	*d60d60GalGal*					5	9	5	5	1											11	11.1	1
			1	1	2	7	9	2		2	1												
					4	5	6	5	3	2													
			1	4	6	7	5	1	1														
				1	2	8	8	1	2														
Koshihikari type (tall and approx. 30% sterile)	*D60d60Galgal*						1						1	6	5	7	2	2			24	22.2	2
													(1)		(1)	(1)	1						
				2			3	1			1		3	5	6	4	1						
													(1)		(2)								
									1		1		1	1	3	7	5	2	2				
															(2)	(3)	(1)	(1)					
					1	1			2	1	3	2	1	3	3	7	1	1					
												(1)		(3)									
			1		1					1	3	1		3	2	6	5	3					
														(1)		(2)	(3)						
Koshihikari type (tall)	*D60d60GalGal*				5			1	1				1	2	5	6	3	1			19	22.2	2
					1	2		2	1	2	1		3	2	6	4	2						
						1		3	1	1				3	2	3	5	4	2				
				1	1	1	1	1			1	3	3	4	3	3	3						
					1		2				2	1	5		5	4	3	2					
	*D60D60GalGal* *D60D60Galgal* *D60D60galgal*											3	5	1	6	3	3	2	2		46	44.4	4
												1	2	9	8	5		1					
													2	2	8	3	5	2					
											1	3	3	4	8	5	1		1				
													1	5	8	6	4	1					
Total																					100	100	
Test for two-gene segregation (1:2:2:4): Χ^2^ = 0.67, 0.80 < *p* < 0.90																							

1) Each lane shows No. of plants in a F_4_ line. Figures in parenthesis shows No. of partially sterile plants.

**Table 2:** Classification of F_4_ lines of segregating type Ⅱ based on segregation of culm length.

Phenotype and Genotype of F_3_		Frequency Distribution of Culm Length and Seed Fertility in F_4_ Lines																			F_4_ Lines		
		Culm Length (cm)																			Observed No.	Expected	
			50					60					70					80				No.	Ratio
Hokuriku 100 type (semidwarf)	*d60d60GalGal*		1	1	1	13	4	3	1				1								24	25.0	1
					6	7	6	1	5														
			1	3	4	7	8	1															
					1	2	9	4	4	3	1												
			1	3	4	7	5	4	1														
Koshihikari type (tall)	*D60d60GalGal*				2	2	1	1		2		2	3	2	6	3	1				49	50.0	2
					2	1	4	1			2	2	5	3	2	2		1					
			1			1	1	1	1			2	1	5	3	7		1					
						1	2			1		1	6		6	3	2	1	2				
			2			2	1					3	6	3	4	5							
	*D60D60GalGal*												1	1	5	8	7	1	1		27	25.0	1
													2	8	7	5	2	1					
													3		3	7	5	3	3	1			
														6	5	6	4	2	2				
												1	2		5	8	5	4					
Total																					100	100	
Test for one-gene segregation (1:2:1): Χ^2^ = 0.22, 0.80 < *p* < 0.90																							
